# Editorial: Interaction of Pathogenic *Escherichia coli* With the Host: Pathogenomics, Virulence and Antibiotic Resistance

**DOI:** 10.3389/fcimb.2021.654283

**Published:** 2021-03-31

**Authors:** Tânia A. T. Gomes, Ulrich Dobrindt, Mauricio J. Farfan, Roxane M. F. Piazza

**Affiliations:** ^1^ Departamento de Microbiologia, Imunologia e Parasitologia, Escola Paulista de Medicina, Universidade Federal de São Paulo, São Paulo, Brazil; ^2^ Institute of Hygiene, University of Muenster, Muenster, Germany; ^3^ Laboratorio Clínico, Hospital Dr. Luis Calvo Mackenna, Santiago, Chile; ^4^ Departamento de Pediatría y Cirugía Infantil, Hospital Dr. Luis Calvo Mackenna, Facultad de Medicina, Universidad de Chile, Santiago, Chile; ^5^ Laboratório de Bacteriologia, Instituto Butantan, São Paulo, Brazil

**Keywords:** *Escherichia coli*, virulence factors, genome, antibiotic resistance, evolution

*Escherichia coli* live as commensals in the intestines of humans and warm-blooded animals (Leimbach et al., 2013). Although most *E. coli* strains rarely produce disease in healthy individuals, pathogenic strains can cause a wide range of diseases in the gastrointestinal tract or extraintestinal sites in healthy and immunocompromised individuals (Kaper et al., 2004). This variety of behaviors is due to the existence of virulence genes in mobile genetic elements and the large permissiveness of *E. coli* to acquire these elements by horizontal gene transfer (Dobrindt et al., 2004; Kaper et al., 2004; Croxen and Finlay, 2010; Leimbach et al., 2013; Johnson and Russo, 2018).

The acquisition of specific combinations of virulence genes defined the presently recognized diarrheagenic *E. coli* (DEC) pathotypes, which constitute the most critical contributors to diarrhea cases, especially in infants and young children in low- and middle-income countries (Gomes et al., 2016; Jesser and Levy, 2020). These pathotypes differ concerning their virulence mechanisms, preferential sites of intestinal colonization, symptoms, and clinical presentation. In turn, *E. coli* strains involved in extraintestinal infections are collectively known as extraintestinal pathogenic *E. coli* (ExPEC), as their virulence factor arsenal allows their spread to and multiplication in extraintestinal organs, leading to signs and symptoms mainly in the urinary tract, blood, and meninges (Russo and Johnson, 2000; Vila et al., 2016; Biran and Ron, 2018).

Although clinical outcomes may vary in severity, pathogenic *E. coli* remains a public health concern as they continue to gain novel traits, occasionally resulting in more virulent strains. This Research Topic highlights our growing understanding of the process of host-pathogen interactions as it relates to *E. coli*, addressing the genetic diversity, evolution, antimicrobial resistance, and novel molecular mechanisms and virulence strategies in their interaction with the host in various disease conditions. *E. coli* typing, diagnostic, and potential therapy procedures are also discussed.

## Novel Mechanisms of Pathogenicity in The *E. coli* pathotypes

Shiga toxin-producing *E. coli* (STEC) and its subgroup, enterohemorrhagic *E. coli* (EHEC), is one of the most relevant DEC pathotypes. STEC/EHEC strains can cause diarrhea, hemorrhagic colitis in humans, and occasionally lead to hemolytic uremic syndrome (HUS) (Gianantonio et al., 1968; Boyce et al., 2002) and encephalopathy (Obata, 2010), which can be reversible or permanent (Melton-Celsa et al., 2012). STEC colonize the intestines and produce Shiga toxin 1 (Stx1) and/or 2 (Stx2) (Melton-Celsa, 2014), which are released into the intestinal lumen, translocated to the circulatory system, and then bound to their receptor, globotriaosylceramide (Gb3) (Zumbrun et al., 2010), in target cells. However, toxin uptake exhibits both a Gb3-dependent (Sandvig et al., 2002) and a Gb3-independent binding depending on the context (Malyukova et al., 2009; Lukyanenko et al., 2011; In et al., 2013). In this Research Topic, two relevant models, intestinal and microglial cells (MG), are presented to address the pathways of Stx2 uptake. Garimano et al. demonstrate that a hypervirulent O157:H7 STEC strain increases the Stx2 cytotoxic effect by stimulating several endocytic pathways and enhancing Stx2 translocation across HCT-8 monolayers in both the paracellular and transcellular pathways, employing dynamin-independent and Gb3-dependent mechanisms. Berdasco et al. hypothesize that Stx2, either the holotoxin or the Stx2B subunits, exerts a direct biological effect on MG. To determine whether culture conditions affect MG cell sensitivity and responsiveness, they analyze functional parameters; and demonstrated that MG cells exhibit both Gb3-independent and Gb3-canonical pathways for Stx2 uptake and have a pivotal role in the inflammatory processes observed in clinical HUS encephalopathy.

In addition to the production of Stx1, Stx2, and other virulence factors, recent studies revealed that EHEC can produce a type VI secretion system (T6SS) essential to disease development in a murine model (Wan et al., 2017), which has been associated with a higher prevalence of HUS. Vazquez-Lopez and Navarro-Garcia investigate *in silico* the EHEC T6SS core proteins and putative effector and immunity proteins. They compare the corresponding genes between two published genomes of the prototype EHEC O157:H7 strain EDL933 and with the genome of other O157:H7 strains. Unlike other typical T6SS *E. coli* effectors, the authors identify several Rhs family genes (recombination hotspot) (Bondage et al., 2016) in EHEC that could behave as T6SS effectors. These genes could serve as immunity proteins since they have several similar interaction motifs and structural homology with other known immunity proteins.

Of the six classical DEC pathotypes, enteroaggregative (EAEC) and diffusely adherent *E. coli* (DAEC) are the least characterized. To date, not a single DAEC genome and only a few different EAEC genomes have been sequenced. Meza-Segura et al. sequenced the whole genome of ten DAEC and ten typical EAEC strains (positive for the *aggR* gene, which encodes a transcriptional activator of EAEC virulence-associated genes) from diarrheic patients and one commensal *E. coli* strain isolated from a healthy child. They showed that DAEC and typical EAEC are phylogenetically related, but strains of the different pathotypes harbor genes encoding for different sets of virulence factors; DAEC carry more genes encoding for iron acquisition factors, while typical EAEC harbor genes encoding toxins and bacteriocins. Interestingly, the authors identified associations between the clinical characteristics of the diarrheal episode and specific virulence gene profiles in DAEC and typical EAEC.

Dias et al. present an extensive characterization of the typical and atypical EAEC. These authors characterized 220 EAEC isolates obtained from diarrheal patients during seven years (2010-2016) of epidemiological surveillance in Brazil. These isolates were classified into distinct phylogroups, with most isolates assigned to phylogroups A or B1. Interestingly, genes encoding aggregate-forming pili (AFP) were exclusively detected in atypical EAEC, representing a putative novel marker for increasing the efficiency of atypical EAEC diagnosis.

Virulence gene expression is a highly regulated process, mediated by environmental conditions and/or bacterial regulators, which can induce or silence its expression (Kitamoto et al., 2016). Under well-defined environmental conditions, virulence gene expression occurs at a specific site, allowing bacteria to initiate the infection process (Carlson-Banning and Sperandio, 2018). Gut microbiota, or its metabolites, play a fundamental role in regulating pathogenic mechanisms and colonization resistance (Vogt et al., 2015; Rolhion and Chassaing, 2016). In a pilot study, Gallardo et al. determine the composition of gut microbiota and metabolome in stool samples obtained from healthy children and children with diarrhea positive for DEC pathotype. Interestingly, a differential metabolome and microbiota composition was identified between these groups. Additionally, a strong correlation between a gut microbiota species and specific metabolites, such as histamine and L-ornithine, was found in the DEC group; such information might help identify mechanisms and signaling molecules involved in the crosstalk between microbiota and DEC pathotypes.

Besides the production of a variety of virulence factors, uropathogenic *E. coli* (UPEC) strains may produce the vacuolating autotransporter toxin (Vat), which is one of the so-called serine protease autotransporter proteins of the *Enterobacteriaceae* (SPATEs) toxin family (Henderson and Nataro, 2001; Dutta et al., 2002; Nichols et al., 2016). In an urothelium model of bladder cells, Díaz et al. showed that treatment with Vat resulted in time-dependent vacuole formation and loss of the intercellular contacts, leading to changes in the monolayer permeability with a limited amount of cell death. Cellular damage also included cytoskeletal alterations in the urothelium and lamina propria of the bladder and loss of integrity of the urothelium in an experimental *ex vivo* murine bladder model. The Vat-specific targets on the epithelial cell surface or the lamina propria, as well as the composition of the Vat-induced vacuoles, remain to be determined to elucidate the contribution of this toxin to UPEC pathogenesis.

*E. coli* and other members of the *Enterobacteriaceae* may produce siderophore-microcins, which are peptides with antimicrobial activity that, by mimicking the iron–siderophore complexes, penetrate and kill phylogenetically related bacteria (Duquesne et al., 2007). Massip and Oswald provide an overview of the recent understanding of the siderophore-Mcc genetic determinants and biosynthesis, their mechanisms of action, and biological relevance in *E. coli*. They also show that the UPEC siderophore-microcin gene clusters and biosynthetic pathways differ from the “archetypal” types of fecal *E. coli* strains. Production of an active siderophore-microcin depends on the synergistic action of proteins encoded in other genomic islands and confers a strong selective advantage to control the colonic niche.

Rueter and Bielaszewska review the exciting topic of outer membrane vesicles (OMVs) production by Gram-negative bacteria, emphasizing intestinal pathogenic *E. coli*. OMVs are nanoscale proteoliposomes secreted from the cell envelope (Amano et al., 2010; Ellis and Kuehn, 2010; Kulp and Kuehn, 2010; O’Donoghue and Krachler, 2016). They represent a highly advanced mechanism for secretion and delivery of bacterial virulence factors into host cells, improving bacterial fitness, and supporting bacterial interactions with polymicrobial communities and the host (Manning and Kuehn, 2011; Duperthuy et al., 2013). OMV production contributes significantly to bacterial virulence since it makes it more able to reach and colonize distant host tissues, impair cell functions, and modulate the host’s defenses. Therefore, efforts have been made to exploit the antigenic and adjuvant properties of OMVs as promising vaccine components. However, much knowledge is required to define the immunogenicity and protective efficacy of OMVs and to identify their components involved in the immune responses and mechanisms underlying OMV-elicited protective immunity.

## Diagnosis, Typing, and Genomic Evolution in the *E. coli* Pathotypes

Molecular diagnostics is becoming increasingly important to allow detection and diagnosis of pathogens also culture-independently. They are interesting for medical applications and fundamental questions, e.g., regarding the prevalence and ecology of pathogens and food safety (Ramanan et al., 2017; Sekse et al., 2017). For example, the current focus of routine STEC detection is still on the predominant “big seven” serotypes associated with clinical symptoms. Nevertheless, comprehensive diagnostic tests for all clinically relevant STEC serogroups are required. Ludwig et al. took advantage of available genomic sequence information of STEC O antigen-specific genes and developed and validated multiplex PCR (mPCR) assays for the discrimination and detection of 137 “non-big seven” STEC serogroups, which can be associated with cattle. These mPCR assays can, for instance, help to systematically screen the prevalence of STEC in the environment or animals.

Merino et al. applied real-time quantitative PCR (qPCR) to analyze in a culture-independent way the prevalence of bacterial enteropathogens in stool samples of children below seven years with and without diarrhea in São Paulo, Brazil. They detected the tested enteropathogens’ virulence markers significantly more frequently in stool samples from diarrhea cases than asymptomatic controls. Also, the relevant marker copy number was significantly higher in diarrheal patients than in stool from asymptomatic children. This analysis demonstrates that asymptomatic children of an urban area, such as São Paulo, may be a reservoir of enteropathogens.

Michelacci et al. analyzed whole-genome sequences of highly virulent enteroinvasive *E. coli* (EIEC) O96:H19 isolates. Sequence comparison of the EIEC virulence plasmid indicated that IS element-mediated recombination might be responsible for the absence of the conjugation determinant in most EIEC and Shigella virulence plasmids. The authors hypothesize that the acquisition of virulence plasmids *via* conjugation led to the evolution of EIEC from non-pathogenic *E. coli* and may promote the establishment of new virulent EIEC clones.

Flament-Simon et al. studied the epidemiological differences of 188 extended spectrum beta-lactamase (ESBL)-producing extraintestinal pathogenic *E. coli* isolates from two hospitals in Spain and France. Although these isolates were markedly diverse, most of them belonged to only three clonal complexes. The new globally emerging clone ST1193 was identified in two isolates from France and Spain in 2015.

*E. coli* is characterized by high genomic plasticity and frequent exchange of genetic material. Thus, unambiguous typing of clinical isolates may be complicated due to the existence of hybrid strains combining different pathotypes’ traits. Comparative genomics is, therefore, instrumental in understanding pheno- and genotypic variability among clinical isolates. Valiatti et al. characterized geno- and phenotypically uropathogenic *E. coli* isolate 252 (UPEC 252) and demonstrated that this strain represents an atypical enteropathogenic *E. coli* (EPEC) strain. The ability to grow in human blood serum and adhere to human epithelial cell lines of the urinary and intestinal tract enables UPEC 252 to cause intestinal and extraintestinal infections.

## Hybrid- and Hetero-Pathogenic *E. coli* Strains

It has been reported that certain pathogenic *E. coli* strains combine different pathotypes’ main virulence traits, encompassing potentially more virulent hybrid strains (Dobrindt et al., 2003; Bielaszewska et al., 2007; Khan et al., 2018). The terms hybrid- and hetero-pathogenic *E. coli* were created to depict new combinations of virulence factors among classic *E. coli* pathotypes. Two mini-reviews on this topic can be appreciated in this Research Topic. Santos et al. review the studies that introduced the hybrid- and hetero-pathogenic *E. coli* classifications, emphasizing the *E. coli* genomic plasticity that emerged in mixed pathotypes exhibiting unique pathogenic mechanisms. The potential of such hybrid strains to emerge in new and severe outbreaks and their potential implication in more severe diseases are also discussed. Braz et al. add an important topic to this discussion, i.e., the increasing acquisition of antimicrobial resistance by *E. coli* strains. The consequences related to this genetically versatile species are the growing need to develop unconventional therapies and more precise diagnostic methods to combat the infections caused by these hybrid strains.

## Novel Procedures for Dec Infections Therapy

Gut microbiota has been associated with resistance to pathogen colonization in the intestine. Several molecules have been proven to modify the composition of the gut microbiota (Pamer, 2016; Jacobson et al., 2018). Liu et al. investigated the changes in gut microbiota induced by *Pulsatilla decoction* (PD), a traditional Chinese medicinal herb used to treat fever and dysentery, which also has a good curative effect on bacterial diarrhea and inflammatory bowel disease. The authors studied changes in gut microbiota after PD therapy of *E. coli *infection in rats and found that PD helped restore *Bacteroidetes* spp. composition in the gut. These findings might be essential to determine the mechanism of the Chinese herbal formula for preventing and treating bacterial infections.

The use of antibiotics to treat STEC infections has long been controversial due to reports that such treatments may increase Shiga toxin secretion (Wong et al., 2000); currently, the recommended therapy is mainly supportive. Mühlen and Dersch reviewed the current understanding and progress in developing treatment options against STEC infections. In recent years, several strategies have progressed to the clinical trial stages. Receptor analogs such as Synsorb Pk, or the use of Eculizumab looked promising in phase II trials but showed little evidence of success when evaluated systematically or in phase III trials (Trachtman et al., 2003; Monet-Didailler et al., 2019). On the other hand, a prophylactic vaccine may only be of interest for countries where these infections are endemic. In general, phase II clinical trials can be carried out, but patients with STEC infections for phase III trials are limited.

## Conclusions

The recent progression of genome sequencing techniques allowed identifying novel virulence factors that enable *E. coli* strains to harm the human host. The *E. coli* genetic plasticity favors the emergence and spread of virulence traits and antimicrobial resistance, resulting in novel virulent variants. These isolates include the so-called hybrid- and hetero-pathogenic strains, which exceed the borders currently established in defining the different *E. coli* pathotypes and represent an emerging threat that challenges the development of novel diagnosis and typing methods. Knowing the different virulence strategies employed by *E. coli* in its interaction with the host in various disease conditions reveals potential new targets for disease prevention and treatment.

## Author Contributions

All authors listed have made a substantial, direct, and intellectual contribution to the work, and approved it for publication. All authors contributed to the article and approved the submitted version. 

## Conflict of Interest

The authors declare that the research was conducted in the absence of any commercial or financial relationships that could be construed as a potential conflict of interest.
